# Behavior of the Siemens Virtual Wedge™ following an interruption to beam delivery

**DOI:** 10.1120/jacmp.v4i2.2526

**Published:** 2003-03-01

**Authors:** N. D. Richmond, C. P. Walker

**Affiliations:** ^1^ Regional Medical Physics Department Newcastle General Hospital Newcastle upon Tyne NE4 6BE United Kingdom; ^2^ Sarah Oncology Centre Hamad Medical Corporation P.O. Box 3050 Doha Qatar

**Keywords:** radiotherapy, Virtual Wedge™

## Abstract

Investigations were made into the beam profile shape and dose delivered by the Siemens Virtual Wedge™ under standard operational conditions compared with those following delivery interruption on two Siemens Primus linear accelerators (Type 7445 and 8067) running different versions of control software (7.2 and 7.0, respectively). The shape of the Virtual Wedge™ profiles was found to be unaffected by beam delivery interruption. An increase in the dose delivered to the central axis was found when delivery was interrupted and subsequently resumed using information recorded in a recall data file on one of the accelerators. This dose increase was attributed to a difference in delivered monitor units recorded in the recall data file compared to those displayed on the linear accelerator control console.

PACS number(s): 87.53.–j, 87.52.–g

The use of wedge filters in radiotherapy to produce dose gradients across the beam profile is widespread. Traditionally this is done using physical wedges made of metallic material shaped in such a way so as to produce graduated attenuation across the radiation beam. Controlling the movement of one of the collimator jaws and the delivered dose rate throughout irradiation can produce similar beam profiles. The Siemens Virtual Wedge™ uses this philosophy to replace physical wedge filters. The theory and operation of the Virtual Wedge™ will not be discussed here for brevity but may be found in some detail in the papers by van Santvoort1 and Rathee *et al.* ^2^

If patient treatment using a physical wedge is interrupted part way through, it is a straightforward process to resume delivery. It is simply a matter of delivering the remaining monitor units through the wedge filter. Small dose delivery anomalies may present themselves due to the rounding of part monitor units delivered during the first portion of treatment. These are likely to generate only small errors in delivered dose. The dose profile in the wedge gradient direction remains unchanged. However, the part delivery of a wedge field that employs dynamic jaw motion e.g., the Siemens Virtual Wedge™, presents different dosimetric problems. The position of the dynamic jaw at the moment of beam termination must be known, together with the number of monitor units delivered, to correctly resume wedged field delivery. The Siemens Primus Linear accelerators contain software allowing for the resumption of any field delivery following such beam terminations. A nonvolatile memory is updated directly from the monitor chamber every 0.1 sec with information enabling the calculation of delivered monitor units. This data is stored in Hex format and can be read out directly from a LCD display located behind the gantry fascia. The Hex reading is converted into monitor units delivered by multiplying by a constant, *k* (also read from the LCD display) and then divided by 1000. Alternatively, the same data can be accessed from the Linac control console using the recall data function. The position of the moving jaw is recorded in a similar fashion within the memory cell. The following two methods of termination and restart of the Virtual Wedge™ were investigated.

(i) The radiation beam delivery was terminated using the “Rad off key” and then restarted without clearing the LINAC control console. This simulates an operator‐interrupted beam with subsequent continuation of radiation delivery. In this scenario the recall data file is not required.

(ii) The radiation beam delivery was terminated using the “Rad off key” and restarted following recourse to the information stored in the LINAC recall data file. This simulates delivery termination due to either machine fault or power loss. Inputting the recalled data (delivered monitor units, initial pre‐set monitor units, and dynamic jaw position) into the LINAC control console enables the resident software to recalculate the dynamic jaw speed and dose rate variation to correctly deliver the remaining portion of the Virtual Wedge™ field. Using the recall data file in this way is the method recommended by Siemens to resume Virtual Wedge delivery.

Investigations were undertaken into the shape of wedge profile produced following an interruption to the wedge delivery sequence by both of the above methods, using film dosimetry. The delivered dose to the central beam axis was also studied with a Farmer ionization chamber under similar conditions. Furthermore, the information contained within the LINAC recall data file was compared to that displayed on the treatment machine console at the point of interruption for method (ii). A comparison was also made between a low energy Siemens Primus (Type 7445) running version 7.2 software and a high energy Primus (Type 8067) with version 7.0 software.

Film dosimetry measurements were made on both Primus LINACs. Kodak Xomat‐V packaged film was sandwiched 10 cm deep in WT1 solid water (Scanplas, St Bartholomew's Hospital, London) with 90 cm source to surface distance (SSD). A $10\times 10\;{\text{cm}}^{2}60{}^{\circ}$ Virtual Wedge™ was delivered at 6 MV. 50 MU were delivered to the central axis of the field, giving a dose of 0.39 Gy to the film. Additional films were exposed with the Virtual Wedge™ delivery interrupted and delivery concluded by the two methods previously described. In each case the beam was terminated after 24 MU had been delivered. The moving collimator jaw was at a position of 2.0 cm past the central axis. Film measurements were also taken for a range of field sizes, monitor unit settings, and at several termination points. No significant difference in wedged profile shape was seen between interrupted and unmolested beam deliveries. However, the optical density of the film taken with the Virtual Wedge™ interrupted and delivery completed using information contained within the recall data file was seen to be greater than that of the just interrupted and uninterrupted wedges across the entire profile on the low energy Primus. This was not the case with the high energy Primus films. A larger dose had been recorded on the film with the recalled Virtual Wedge™ delivery on the low energy Primus. Examples of the film profiles of the Virtual Wedges™ for the low energy Primus showing this effect are plotted in Fig. 1.

**Figure 1:**
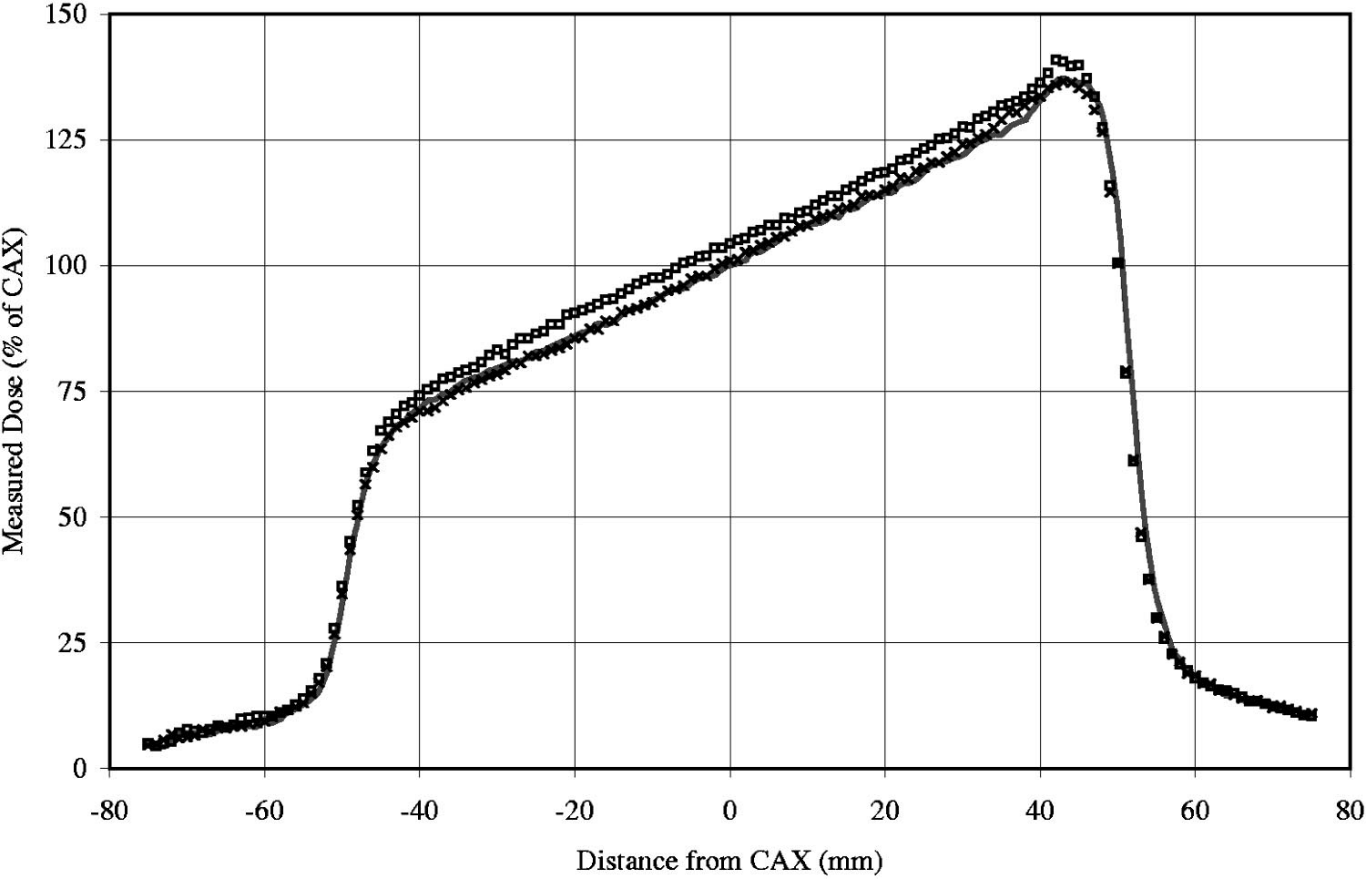
Film profiles at 10 cm deep of three, $10\times 10\;{\text{cm}}^{2},60{}^{\circ}$ Virtual Wedge™ deliveries. Uninterrupted delivery (–), interrupted and restarted (x), and interrupted and restarted using information from the recall data file (□) on the low energy Primus accelerator are shown.

Central axis dose measurements were made with a Farmer type ionization chamber on both linear accelerators. The ionization chamber was placed at 10 cm deep in WT1 solid water phantom and at 90 cm SSD. A $10\times 10\;{\text{cm}}^{2}60{}^{\circ}$ Virtual Wedge™ was delivered with 100 MU being given to the central beam axis, a dose of 0.78 Gy. Figure 2 shows the variation in delivered dose, from that of the uninterrupted wedge, of the interrupted and recalled Virtual Wedge™ deliveries on both Primus LINACs. The data has been plotted as a function of MU delivered before interruption along the abscissa. A total of 37 and 60 MUs have been delivered when the moving jaw reaches the central axis and final position respectively. Figure 2 indicates that interrupting the Virtual Wedge™ delivery and restarting it gives a delivered dose differing from that of the uninterrupted wedge by no more than 0.8% on both treatment machines. Dose measurements for the recalled wedge on the high energy Primus are also within 1.0% of the uninterrupted delivery in all cases. The low energy Primus shows an increasing disparity in dose delivered to the beam central axis as the number of MU delivered prior to interruption increased. Dose differences of up to 4.5% were seen.

**Figure 2:**
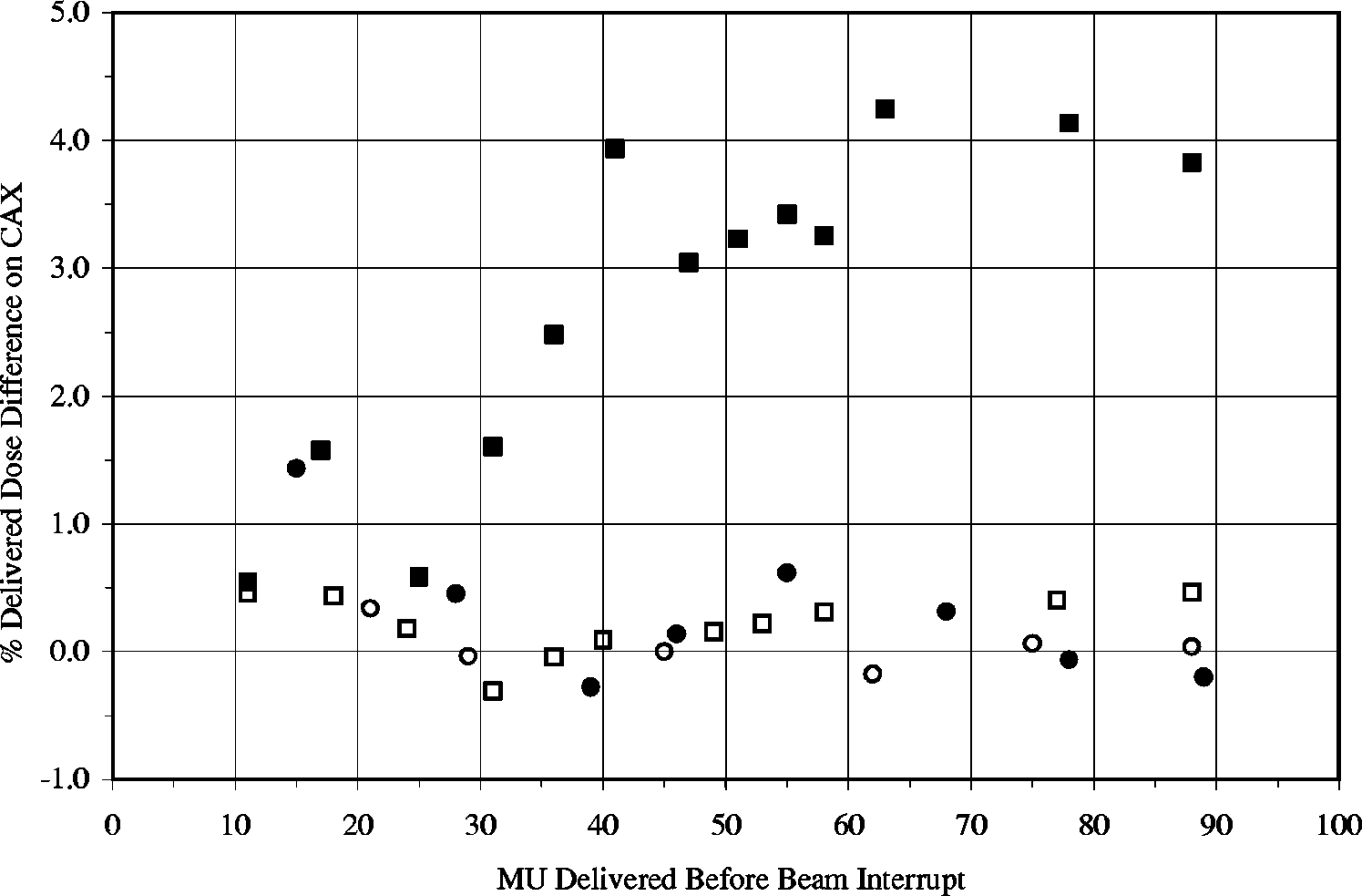
Measured central axis dose differences at 10 cm deep between continuous $10\times 10\;{\text{cm}}^{2},60{}^{\circ}$ Virtual Wedge™ delivery (100 MU) and interrupted delivery on high and low energy Primus accelerators. □, low energy Primus interrupted and restarted; ■, low energy Primus interrupted and recalled from data file; ○, high energy Primus interrupted and restarted; •, high energy Primus interrupted and recalled from data file.

In addition to film and ionization chamber measurements, the information recorded within the LINAC recall data file was compared to that displayed on the LINAC control console at the point of Virtual Wedge™ interruption. The position of the moving jaw at the moment of beam termination was identically recorded in the recall data file and console display screen for both accelerators. There were disparities between the number of monitor units recorded as being delivered on the accelerator console and in the recall data file on the low energy Primus. Those recorded on the accelerator console were correct. This monitor unit difference increased with monitor units delivered prior to termination. The largest discrepancy was found when the beam was terminated at 93 MU delivered according to the LINAC display console, only 90 MU were recorded in the wedge recall data file. Other Virtual Wedge™ field sizes showed similar behavior on the low energy Primus accelerator. Similarly, variation in the central axis monitor units delivered gave comparable interrupted monitor unit differences between console display and recall data file. This was not the case on the high energy Primus, where the recalled monitor units were always identical to those displayed on the LINAC console following Virtual Wedge™ interruption.

The discrepancy between console displayed, and recall data file, monitor unit values on the low energy Primus account for the measured differences in the central axis dose on this treatment machine. The conclusion to be drawn from this data is that, whilst the shape of the wedged field profile is maintained following delivery interruption, the total dose was not necessarily correct. Consultation with Siemens prompted them to issue details of tolerances on parameter backup that will be included in future user manuals. The tolerance for the monitor unit difference between LINAC control console and recall data file is 1% or 2 MU. Clearly this limit was exceeded during our investigations on our low energy Primus. Siemens have acknowledged that the deficiency in recalled monitor unit data was due to a software error and is subject to a corrective action.^3^ Following these measurements, it is considered of vital importance that the functionality of the Virtual Wedge™ resumption software is tested at commissioning and following accelerator control software upgrades.
